# The Incorporation of Etanercept into a Porous Tri-Layer Scaffold for Restoring and Repairing Cartilage Tissue

**DOI:** 10.3390/pharmaceutics14020282

**Published:** 2022-01-26

**Authors:** Yaima Campos, Gastón Fuentes, Amisel Almirall, Ivo Que, Timo Schomann, Chih Kit Chung, Carla Jorquera-Cordero, Luis Quintanilla, José C. Rodríguez-Cabello, Alan Chan, Luis J. Cruz

**Affiliations:** 1 Translational Nanobiomaterials and Imaging, Department of Radiology, Bldg. 2, k4-44, Leiden University Medical Centre, Albinusdreef 2, 2333 ZA Leiden, The Netherlands; Y.Campos_Mora@lumc.nl (Y.C.); I.Que@lumc.nl (I.Q.); T.Schomann@lumc.nl (T.S.); C.K.Chung@lumc.nl (C.K.C.); 2 Biomaterials Center, University of Havana, Avenida Universidad Entre G y Ronda, Vedado, Plaza, La Habana CP 10400, Cuba; amisel@biomat.uh.cu; 3 Bioforge Lab, CIBER-BBN, Campus Miguel Delibes, Universidad de Valladolid, Edificio LUCIA, Paseo Belén 19, 47011 Valladolid, Spain; luisq@ele.uva.es (L.Q.); roca@bioforge.uva.es (J.C.R.-C.); 4 Percuros B.V., Zernikedreef 8, 2333 CL Leiden, The Netherlands; C.A.JorqueraCordero@umcutrecht.nl (C.J.-C.); achan@percuros.com (A.C.); 5 Department of Orthopaedics, University Medical Centre of Utrecht Heidelberglaan 100, 3584 CX Utrecht, The Netherlands

**Keywords:** osteoarthritis, tissue engineering, tri-layer scaffolds, etanercept, implants

## Abstract

Cartilage diseases currently affect a high percentage of the world’s population. Almost all of these diseases, such as osteoarthritis (OA), cause inflammation of this soft tissue. However, this could be controlled with biomaterials that act as an anti-inflammatory delivery system, capable of dosing these drugs over time in a specific area. The objective of this study was to incorporate etanercept (ETA) into porous three-layer scaffolds to decrease the inflammatory process in this soft tissue. ETA is a blocker of pro-inflammatory cytokines, such as tumour necrosis factor alpha (TNF-α) and interleukin 6 (IL-6). For this reason, the scaffold was built based on natural polymers, including chitosan and type I collagen. The scaffold was grafted next to subchondral bone using hydroxyapatite as filler. One of the biomaterials obtained was also crosslinked to compare its mechanical properties with the non-treated one. Both samples’ physicochemical properties were studied with SEM, micro-CT and photoacoustic imaging, and their rheological properties were also compared. The cell viability and proliferation of the human chondrocyte C28/I2 cell line were studied in vitro. An in vitro and in vivo controlled release study was evaluated in both specimens. The ETA anti-inflammatory effect was also studied by in vitro TNF-α and IL-6 production. The crosslinked and non-treated scaffolds had rheological properties suitable for this application. They were non-cytotoxic and favoured the in vitro growth of chondrocytes. The in vitro and in vivo ETA release showed desirable results for a drug delivery system. The TNF-α and IL-6 production assay showed that this drug was effective as an anti-inflammatory agent. In an in vivo OA mice model, safranin-O and fast green staining was carried out. The OA cartilage tissue improved when the scaffold with ETA was grafted in the damaged area. These results demonstrate that this type of biomaterial has high potential for clinical applications in tissue engineering and as a controlled drug delivery system in OA articular cartilage.

## 1. Introduction

Cartilage is a soft tissue located in several areas of the body coating joints. Its main function is to protect joints from cyclic stresses, preventing wear by friction and allowing movement. Some factors, such as mechanical, genetic and biochemical factors, can disrupt chondrocyte–matrix associations and alter metabolic responses in the chondrocytes [1]. This can lead to OA, which is a slowly progressive disease that is very common in the elderly and sportive populations. It is frequently found in the hip, knee, distal phalangeal and intervertebral joints. OA can be primary or secondary [2]. The end stage of this disease is characterised by the deterioration and detachment of the joint bearing and the proliferation of new osteoarticular tissue at the margin. The inflammation caused by OA also affects the subchondral bone in the zone of calcified cartilage, which invades the deep hyaline cartilage layer [3]. It is difficult for cartilage to recover because it lacks lymphatic and blood vessels and it has a scant cellular population. Since their joint function is remarkably affected, the daily living and social activities of patients with OA are restricted.

Several conservative treatments have been used, including intra-articular (IA) injection of a hyaluronan preparation [4]. However, this treatment has some limitations [5]. Orally administered supplements, such as glucosamine and chondroitin sulphate, have also been reported to reduce pain and inhibit the narrowing of the joint space [6]. However, supplements cannot stop the progression of OA. When OA is in its advanced stages, autologous transplantation [7,8] and cartilage cells [9] cannot fight it efficiently [7].

For these reasons, researchers have attempted to mimic the structure and composition of cartilage by the combination of scaffolds, cells and growth factors in the damaged area. This procedure is termed tissue engineering (TE). The TE strategy applies the principles of biology and engineering to the development of functional substitutes for damaged tissue [10]. There are two basic approaches [11]: (1) in vivo TE, in which the construct is implanted with or without prior partial in vitro cultivation and is allowed to mature in vivo for tissue repair and regeneration; and (2) ex vivo TE, in which the tissue is generated entirely in vitro with full functionality before transplantation. In both approaches, the three components that crucially influence the outcome of a TE construct are responsive cells and an appropriate biomaterial and environment.

In this direction, porcine chondrocytes were seeded onto chitosan (CHI) scaffolds with an interconnected porous structure for in vitro study. This system synthesized extracellular matrix at 28 days of culture [12]. Vunjak-Novakovic et. al. developed fibrous polyglycolic acid (PGA) scaffolds by extruding PGA into 13 μm diameter fibres. They were processed into fibrous disks and seeded with bovine articular chondrocytes [13].

Nevertheless, the strong inflammatory process and continuous degradation hindered the complete restoration of this tissue. In the current literature, it is suggested that proinflammatory cytokines, such as interleukin 1 (IL-1) and transforming growth factor- alpha (TGF-α), are involved in the first stages of OA. Synovial cells, such as fibroblast and macrophage-type, as well as chondrocytes are an important source of cytokines, which induce chondrocytes to secrete proinflammatory mediators and the degradation of cartilaginous tissues by proteases.

ETA, a tumour necrosis factor (TNF) antagonist, was approved in January 2002 for the treatment of psoriatic arthritis (PsA). The anti-inflammatory effects of ETA are due to its ability to bind to the pro-inflammatory cytokine TNF, preventing it from interacting with cell surface receptors and rendering it biologically inactive [14].

For that reason, the main goal of this research was to incorporate the drug ETA into a porous multilayer scaffold consisting of natural polymers, including type I collagen and CHI, to diminish the inflammatory process in articular cartilage.

The aim was to mimic the articular cartilage structure and restore a damaged area of this tissue in the knee. The collagen was selected to be used in the structure because cartilage tissue contains several types of this polymer. The chitosan was also incorporated because of its suitable properties: it is biocompatible, biodegradable, mucoadhesive and homeostatic. The ratio of these two natural polymers in each layer was designed to mimic the gradient of composition in the soft tissue. This porous scaffold incorporated calcium phosphate (hydroxyapatite) in the layer next to the subchondral bone to promote the osteogenic character of the material and to allow it to be easily integrated in the damaged area of the articular cartilage. The scaffold was also crosslinked to guarantee its suitable mechanical properties in the grafted area.

## 2. Materials and Methods

All chemical reagents were of analytical grade. They were purchased from Sigma-Aldrich Co. (Madrid, Spain and Amsterdam, The Netherlands) and they were used as received. The source of some other reagents will be explained below.

### 2.1. Hydroxyapatite (Hap)

Calcium phosphate Ca_10_(PO_4_)_6_(OH)_2_, which promotes osteoinduction between the subchondral bone and the biomaterial, was obtained by the humid method. Briefly, an aqueous calcium oxide suspension under stirring (250 rpm) was mixed with a phosphoric acid solution (250 mL, 0.4 mol/L), which was added drop-by-drop as reported in the literature [15].

### 2.2. Polymer Solutions

Two aqueous solutions of natural polymers (type I COL, bovine collagen fibres, commercial grade, Brazil) and chitosan (MW, Sigma Aldrich, Zwijndrechtty, The Netherlands) were prepared at 2% *w/v* as previously reported [16].

Type I collagen is a natural component of skeletal tissues, such as bone tissue. Accordingly, collagen-based scaffolds have certain advantages over other types: for example, they allow for contact between the preloaded cells and endogenous cells located in joint tissues. Collagen-based biomaterials can be fabricated by enriching a collagen solution with biomolecules, such as elastin, chitosan or GAGs. The collagen required for scaffolds can be extracted from biological tissue by using acidic, neutral saline or proteolytic solutions. Interestingly, endogenous proteases can be inhibited during the solubilization of a type I collagen acidic solution. 

Chitosan, a popular constituent material for biomimetic scaffolds, is a partially deacetylated polymeric derivative of chitin, which is commonly found in the cell walls of fungi and in the shells of crustaceans. Chitosan comprises a network of α (1–4)-linked glucosamine units that also contains N-acetyl-glucosamine units. The ratio of glucosamine units to N-acetyl-glucosamine units determines the degree of deacetylation in the polymer, which varies from 30% to 95%. The molecular weight of extracted chitosan ranges from 300 to 1000 kD, depending on its source and the method used for preparation and purification. The solubility of crystalline chitosan in aqueous solutions is pH dependent: above pH 7, it is practically insoluble, but from pH 6 downward, it begins to become soluble due to the protonation of its free amino groups [17].

### 2.3. Preparation of Scaffolds

**Figure 1:**
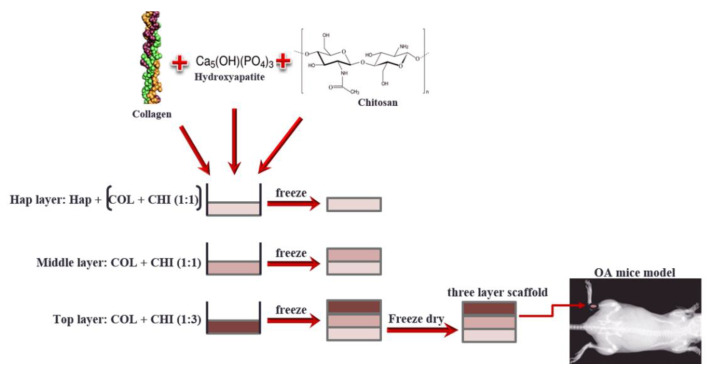
Scaffold preparation scheme.

A tri-layer scaffold was constructed with a graded composition. It had three layers: a bottom, where HAp powder was incorporated, middle and top layer (Figure 1). The natural polymer concentrations were varied with the main purpose of mimicking the articular cartilage. Their porosity was also induced to promote the interchange and movement of fluids and cells inside the material. 

To obtain the bottom layer (B-layer), a mixture with similar volumes of chitosan and collagen solutions (both at 2%) was made by stirring at 5000 rpm with an Ultra-Turrax^®^ stirrer. Later, 0.5% Tween 80 (by mL of layer suspension), 50 µL of near-infrared (IR 780 iodide, 10 mg/mL) and HAp powder (2% (*w/v*) of the final volume) were added. It was stirred for 30 min. Before the stirring was completed, 0.8% of NaOH 1 mol/L (per mL of layer suspension) was added to neutralize the acidic pH. Finally, the suspension was added to a plastic mould and frozen at −80 °C for 30 min.

The procedure and the volume of the polymer solutions ratio used to obtain the intermediate layer (I-layer) were the same, except the calcium phosphate powder was not added. For the top layer (T-layer), the only change made was the natural polymer solutions ratio (75% chitosan/25% type I collagen). At the end, the tri-layer scaffold was freeze-dried in an Alpha 1–4 LD plus freeze-dryer (Martin Christ; Osterode am Harz, Germany).

### 2.4. Crosslinking with EDC/NHS

The EDC/NHS concentration was selected after considering previous works in which the crosslinking agents, their concentrations and the type of polymers in the porous scaffold layers were changed [18]. The best results were obtained for a scaffold based on chitosan/collagen and the crosslinking agents EDC/NHS in a ratio of 33 mmol/6 mmol. Half of the scaffolds obtained were kept in an 95% ethanol solution of EDC/NHS (33 mmol/6 mmol) at room temperature for 4 h. Then, the crosslinked scaffolds were washed with distilled water several times, frozen at −80 °C and lyophilized [19].

### 2.5. Doping with ETA

Scaffolds with and without crosslinking treatment were coupled with ETA. A total of 27 mm^3^ of the cubed dimension of the biomaterials were injected with a solution of the drug to obtain a final concentration of 60 µg/mL for the in vitro study. Later they were frozen at −80 °C and freeze-dried. For the in vivo test, the final concentration selected for the scaffolds with dimensions of 1 mm^3^ was 0.9 µg/mL, following a previous study [20].

### 2.6. Physicochemical Characterization of the Three-Layer Scaffolds

Scanning electronic microscopy (SEM). The surface morphology of the scaffolds and their cellular distribution were evaluated using a NanoSEM 200 microscope (FEI, Hillsboro, OR, USA). The biomaterials were coated with an ultrathin layer (300 Å) of Pd/Pt in an ion sputter coater (Cressington 208HR, Watford, UK).

Micro computed tomography (micro-CT). The scaffold samples were scanned with the high resolution microtomography SkyScan 1076 (Bruker Micro-CT, Kontich, Belgium) at a voltage of 40 kV and a current of 250 μA using 0.5 mm aluminium filter. The same procedure was reported in [16].

Photoacoustic imaging of scaffolds. The scaffolds were placed in an ultrasound gel-containing Petri dish and completely covered with ultrasound gel. Next, the Petri dish was affixed on the imaging table of a Vevo 3100 Imaging System (FUJIFILM VisualSonics, Amsterdam, The Netherlands) with tape. The Vevo 3100 Imaging System was further equipped with a Vevo LAZR-X cart, a Vevo LAZRTight Enclosure and a Vevo Imaging Station. For ultrasound and PA imaging, the MX550D transducer (bandwidth: 32–55 MHz; centre transmit: 40 MHz; axial resolution: 40 μm; imaging depth: −15.0 mm) (FUJIFILM VisualSonics, Amsterdam, The Netherlands) was used in combination with a 14 mm narrow (green) optical fibre.

The scaffolds were visualized by means of ultrasound imaging. Near-infrared (insoluble IR iodide) was excited at 780 nm, as the PA-mode spectrum analysis between 680 nm and 970 nm (in 5 nm steps) revealed optimal signal strength at this wavelength. The PA signal was measured with the MX550D transducer and 3D images were acquired with the Vevo Imaging Station. The acquired photoacoustic data were processed using Vevo LAB 3.2.0.

### 2.7. Rheological Properties

The rheological properties of the samples were measured in a stressed rheometer AR 2000EX (TA Instruments, Newcastle, DE, USA). The samples were wetted on a plate at 37 °C after being previously immersed in MilliQ water in an oven for 15 min. The scaffolds’ cylinders were placed between non-porous parallel rustless-steel dishes (Ø = 12 mm). A normal adequate force to handicap slippage was applied. The opening was greater than 1000 µm until equilibrium was obtained. Firstly, several oscillatory measures were made in the dynamic shear deformation mode. The viscoelasticity’s linear region was determined, where a dynamic sweeping (with tensile efforts between 0.01 and 10%) at 1 Hz frequency was used to measure the dynamic shear module as a function of tension. Secondly, the dynamic testing of frequencies sweeping between 0.01 and 10 Hz (with fixed tensile effort at 0.02%) was used to determine the influence of the dynamic shear module on the storage and loss modulus. Finally, the temporary evolution of the relaxation module was determined. Although it was measured from zero, the normalized values were reported beginning at five seconds. At earlier times, there was a great deal of dispersion. This was to enable the material to adapt to the application of an oscillatory constant force for a period of 1800 s (30 min), selected to determine the long-term behaviour of the scaffold [21,22]. In short, the rheological measures supplied the storage or elastic module (G′), and the loss or viscous module (G″). The loss factor (tan δ) and the phase angle (δ), lag’s angle between the applied stimuli and the corresponding answer were calculated from both moduli. In addition, the complex module (also called the dynamic module of cut effort or shear dynamic module, |G*|^2^ = (G′)^2^ + (G″)^2^) and the transient evolution of the relaxation module G(t) were calculated [23].

### 2.8. Study of the ETA Chemical Bonds in the Scaffold Structure

The scaffold was stained with the primary antibody chicken α-etanercept (Invitrogen, Eugene, OR, USA) and secondary antibody Alexa goat α-chicken 488 (Invitrogen, Eugene, OR, USA) in cryosections of 10 µm of diameter and 3D scaffolds (3 mm width × 3 mm length × 1 mm height) according to the referenced protocol. The materials, positive controls and negative controls were analysed using a Leica DM5500 B fluorescence microscope (filter settings: FITC and DAPI), equipped with a Leica DFC365 FX digital camera. Digital images were acquired and stored using Leica Application Suite X (LAS X) software. All images were subsequently processed using Adobe^®^ Photoshop^®[^ CC software (version 2014.2.1) [24].

### 2.9. In Vitro Cell Viability

*MTS assay*: To study the cytotoxicity of the biomaterials, an MTS assay was performed. The scaffold samples (3 mm width and length × 1 mm height) were loaded with C28/I2 human chondrocytes (1 × 10^4^ per well in 500 µL of DMEM) and incubated for 24, 48 and 72 h. At every time point, the system scaffold cells were incubated with 3-(4,5-dimethylthiazol-2-yl)-5-(3-carboxymethoxyphenyl)-2-(4-sulfophenyl)-2H-tetrazolium (MTS) solution in the dark for 3 h. A total of 100 µL of the supernatant solution was transferred to a 96-well plate, and read at 490 nm of absorbance in a spectrophotometric microplate reader (VersaMax, Dillsburg, PA, USA) (Program Softmax Pro 1.0).

Viability: The capacity of the crosslinked scaffold to allow the growth and proliferation of the immortalized human chondrocytes (C28/I2 cell line) was studied [25]. Briefly, 100 µL with 3 × 10^4^ chondrocytes per well was seeded on the top of the specimens (3 mm width and length × 1 mm height) with DMEM in a 48-well plate. After 3 h of incubation and cell adhesion, 300 µL of medium was added. The scaffolds were removed after 3, 7 and 14 days of seeding, and later assessed using a calcein-AM/ethidium homodimer-1 (EthD-1) LIVE/DEAD^®^ kit, according to the manufacturer’s instructions. In the LIVE/DEAD^®^ assay, living cells are stained green and dead cells are stained red [26].

### 2.10. In Vitro Anti-Inflammatory Evaluation of ETA

Nitric oxide production: RAW 264.7 macrophages (InvivoGen, San Diego, CA, USA) were cultured in a 96-well plate with or without scaffolds either loaded with ETA (20 µg/mL) or without, together with or without 1 µg/mL LPS for 24 and 48 h. The amount of stable nitrite was assessed by means of a colorimetric assay using the Griess reagent (Promega, Madison, WI, USA), which detects the final NO product generated by activated macrophages and released into the growth medium. Briefly, 50 µL of culture supernatant was mixed with an equal volume of Griess reagent and incubated at room temperature, in the dark for 10 min. The absorbance of the resultant dye was measured at a wavelength of 550 nm in a microplate reader (VersaMax, Dillsburg, PA, USA) with SoftMax Pro software. The nitrite concentration was determined by extrapolation from a sodium nitrite standard curve.

IL-6 and TNF-α secretion: RAW 264.7 macrophages were cultured in a 96-well plate with or without scaffolds either loaded with ETA (20 µg/mL) or without, together with or without 1 µg/mL LPS for 24 and 48 h. Supernatants were obtained and frozen at −80 °C until analysis. The presence of IL-6 and TNF-α in the culture medium was determined by an enzyme linked immunosorbent assay (ELISA) kit according to the manufacturer’s instructions (BioLegend, San Diego, CA, USA). Both TNF-α and IL-6 were measured in triplicate, and ELISA plates were read using a microplate reader (VersaMax, Dillsburg, PA, USA) with SoftMax Pro software 1.0.

### 2.11. In Vitro ETA Controlled Release Study

The in vitro ETA controlled release study was carried out in 200 μL of PBS solution. The system scaffolds—ETA crosslinked or not crosslinked—were kept at 37 °C for 14 days. The whole volume was collected and replaced by fresh PBS solution every 30 min for 6 h. After this time point, the measurements were made every 60 min for 7 h. From that moment on, the volume was collected at 24, 48, 72 h, 7 and 14 days. Finally, the absorbance of ETA in the solution was measured (¦Ë = 280 nm, slope = 1.18 mL/mg, R^2^ = 99.77%) by a Thermo Scientific NanoDrop 1000 One Microvolume UV-Vis Spectrophotometer. Due to the hydrophilic nature of the material, all data were fixed using the Peppas power law (M_t_/M_∞_ = kt^n^). This is because if the liberation was completely controlled by diffusion, the value of n would be 0.5; if not, we would discuss the liberation of another mechanism, including the option of multiplying an influence.

### 2.12. In Vivo Study

Animal model. The in vivo study was performed complying with the Dutch National Law on animal experiments, after the approval of the research protocol by the LUMC Animal Welfare Committee (Register: PE.18.101.002, AVD.: AVD1160020171405, 1 July 2019). To evaluate the anti-inflammatory effect of the scaffolds alone and in combination for cartilage regeneration in vivo, a total of 23 male C57BL/6Jico 12-week-old mice were purchased from Charles River, France. Five of them were kept healthy as negative controls and 18 were injected with collagenase in the left knee to achieve chemically-induced OA (Leahy et al., 2015). Six empty scaffolds and six scaffolds with ETA (1 mm^3^ cubes) were grafted into the OA mice; six OA mice were also injected with ETA solution. Then, they were studied for in vivo stability over 35 days. In brief, the mice were operated on under 2% isoflurane anaesthesia, and a lateral incision (≈1 cm) was made in the left knee; the joint capsule was exposed longitudinally. The scaffolds were carefully slid into the space of the articular cartilage, and then the wound was sutured. The mice were sacrificed at day 35 post-implantation; their hind limbs were removed and studied ex vivo by safranin-O and fast green staining.

In vivo ETA controlled release study. The controlled release study of ETA was performed by collecting 10 µL of blood at 0, 1, 2, 3, 6, 9 and 12 days for 2 min. The serum was separated and kept at −20 °C. The ETA concentration was determined by an ELISA assay, interpolating the values of the dilutions in the standard curve.

In vivo micro-CT scan. Micro-CT scans were performed under injection anaesthesia (100 mg/kg ketamine + 12.5 mg/kg xylazine). They were carried out using a SkyScan 1076 Micro-CT scanner (SkyScan, Kontich, Belgium) with the source voltage and current set to 50 kV and 200 μA, respectively, and an X-ray source rotation step size of 1° over a trajectory of 180°. The CT scan images of the mice legs were taken with an image pixel size of 18 μm and the bones were scanned at the end with an image pixel size of 9 μm. All scans had a frame with an average of three to reduce noise. Reconstructions were performed using the nRecon V1.6.2.0 software (SkyScan) with the beam hardening correction set to 10%, the ring artifact correction set to 10 and the dynamic range set to −1000–4000 Hounsfield units.

In vivo NIRF imaging technique. Scaffolds with near-infrared fluorescent dye IR-780 in the middle layer were imaged by NIRF after implantation in the OA joint using the PEARL IMPULSE imaging system (Li-Cor, Lincoln, NE, USA). The data were analysed using the IMPULSE software. Mice were anesthetized with isoflurane balanced with oxygen during the image acquisition, and each imaging session was performed in less than 1 min. The animals were sacrificed by cervical dislocation at the end of the experimental period, on day 35.

### 2.13. In Vivo Study

The histological study of the mice’s knees was carried out in paraffin cryosections of 10 µm in diameter. They were previously treated and later stained with safranin-O, according to the referenced protocols [27]. The sections were then analysed with a Zen microscope (Zeiss Axio Scan.Z1, equipped with a Colibri LED light source, Hitachi BF colour camera and an ORCA-Flash sensitive camera for fluorescence imaging). The images obtained were stored and processed with the Zen 3.3 software (blue edition).

### 2.14. Statistical Analysis

Except for the in vivo experiment, all assays were performed in triplicate. All statistical analyses and graphing were performed with Origin 2021 software (OriginLab Corp., Northampton, MA, USA). Data are reported here as mean ± standard deviation (SD), unless otherwise stated. Data were analysed using Students’ *t*-test and two-way analysis of variance (ANOVA). In all analyses, significant difference was inferred at α = 0.05.

## 3. Results and Discussion

A porous three-layer bioscaffold based on natural polymers, including chitosan and type I collagen, was designed and constructed to restore damaged articular cartilage. The gradient of the polymer’s ratio was established to mimic this natural tissue. A calcium phosphate (HAp) was incorporated in the layer next to the subchondral bone as well [16].

### 3.1. Physicochemical Characterization of the Scaffolds

**Figure 2:**
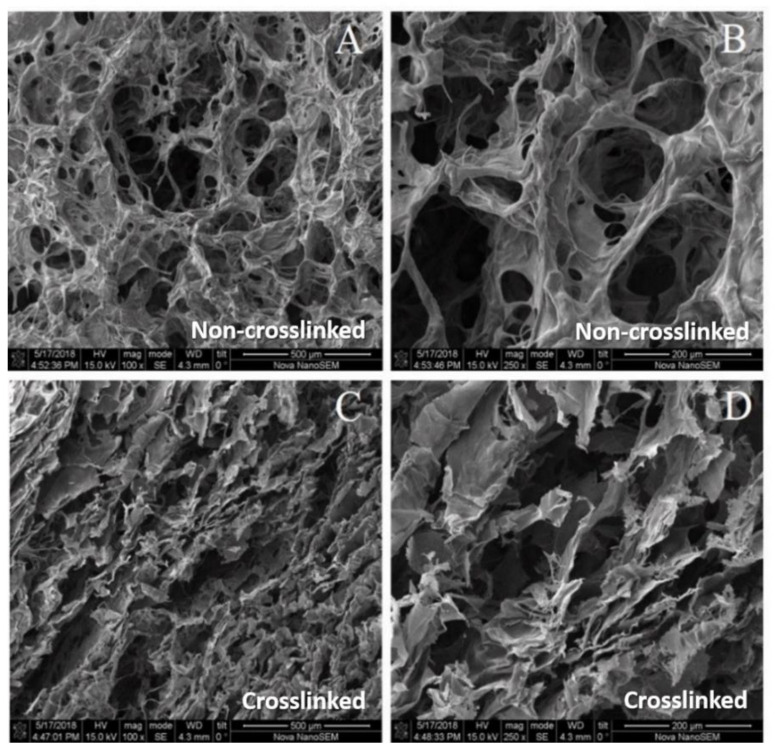
Scanning electron micrographs of the material’s surface. (**A**) Micrograph of the scaffold at 100×, revealing the high porosity throughout the surface. (**B**) Micrograph of the scaffold at 250×, showing the connections between internal pores. (**C**) Micrograph of the crosslinked scaffold at 100×. (**D**) Micrograph of the crosslinked scaffold at 2500×, demonstrating the difference of the morphology when the biomaterial is treated.

The morphology of the scaffold must be porous in order to allow the interchange of fluids, nutrients and cells once it is grafted in the damaged area of the knee. However, the structure cannot be too open because the biomaterial has to support the cyclic mechanical charge without failing. For this reason, the scaffold was crosslinked and its morphology was compared with the non-crosslinked material. Figure 2A,B visibly show that the material had a high concentration of pores (average size = 54 ± 5 µm) due to the action of the surfactant (Tween 80) when the material was being manufactured. This macroporosity is suitable for the adhesion and proliferation of chondrocytes, which are between 5 and 15 µm of size [28]. In Figure 2B, which has a higher magnification, the interconnection of the pores is clearly observed. This characteristic guarantees a better interaction between the graft and the surrounding medium. This allows cell adhesion inside the biomaterial because the chondrocytes can be driven by the DMEM medium through the porous network to adhere, grow and proliferate inside the matrix; otherwise, the cells will just remain on the surface. Additional scaffold characteristics that help with cell attachment, such as its rugosity and polymeric components, make this biomaterial a suitable host for chondrocytes.

The Figure 2C,D show a more compact and dense structure of the treated material compared to the non-treated material. This was expected because there must be a compromise between the porosity and the mechanical properties. The pore sizes could not be calculated by Image J software due to the irregularity of their shape; however, they were interconnected and they had a significant macroporosity, as shown in Figure 1D (higher magnification).

### 3.2. Micro-CT, Optoacoustic Imaging and Scanning Electron Microscopy

**Figure 3:**
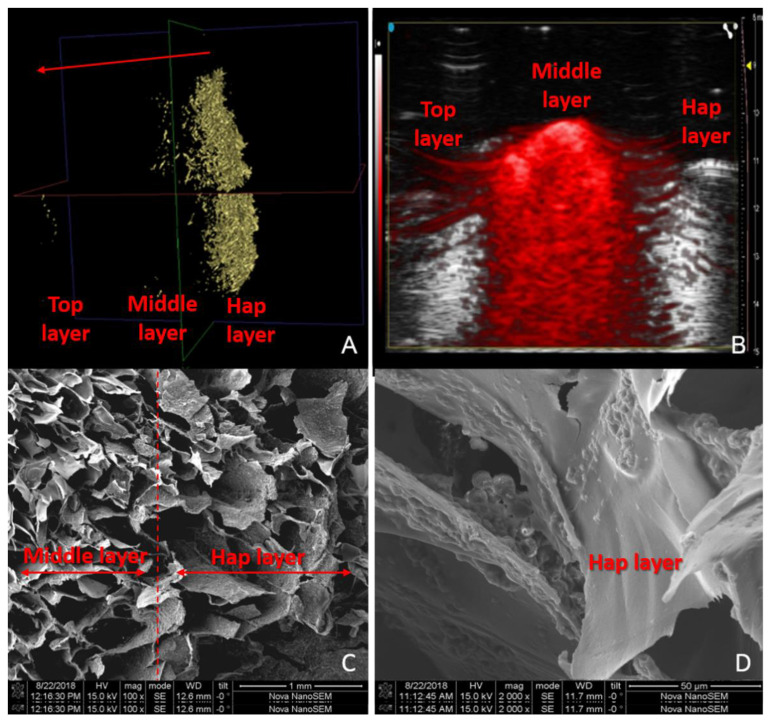
(**A**) Micro-CT of the scaffold showing the hydroxyapatite in the bottom layer. (**B**) Optoacoustic image of the scaffold showing the NIR fluorescence in the middle layer. (**C**) SEM of the scaffold section in which the middle (polymeric) and the bottom (calcified) layers are separated by a red dashed line. (**D**) SEM at higher magnification.

Micro-CT is a 3D imaging technique that utilizes X-rays to see inside an object slice-by-slice. For the scaffold, this technique was used to ascertain the location of the inorganic filler (HAp) in the matrix immediately after the freeze-drying process and several washes. Figure 3A shows that almost 100% of the hydroxyapatite was located in the bottom layer; a few particles migrated to the middle layer but not a representative quantity. This was appropriate because the function of the calcium phosphate was to guarantee the induction of bone with subchondral tissue, and for the bottom layer to be located next to this calcified zone [16].

Optoacoustic imaging is a biomedical modality based on the photoacoustic effect. The optical stimulation of acoustic signals enables multi-scale, high-resolution, non-invasive imaging of both structure and function deep into tissue. The middle layer of the scaffold was loaded with NIR fluorescence followed by the use of imaging techniques in the in vivo experiments. In this direction, the dye was observed by photoacoustic techniques to check its location and intensity inside the material. Figure 3B shows that the dye is exactly in the middle layer of the scaffold and it has a high intensity (red), which may help with the successful monitoring of the biomaterial in the knee. Some authors have also used this technique to characterize scaffolds and differentiate biomaterials from tissue and cells [29].

Figure 3C clearly shows the morphological difference between the layers: the polymeric layer had a smooth surface while the calcified layer was rough due to the apatitic powder. Despite this, both layers were not separated by a gap, guaranteeing that the material will not fail quickly under cyclic stresses. The polymeric and the calcified layer differed some in terms of their porosity and pore size. This characteristic is also appropriate because the subchondral bone cells are osteoblasts, which are between 20 and 50 µm and bigger than chondrocytes. Figure 3D presents a magnification of a zone where both layers coexist, and all the details of the surface can be observed.

### 3.3. Rheology

**Figure 4:**
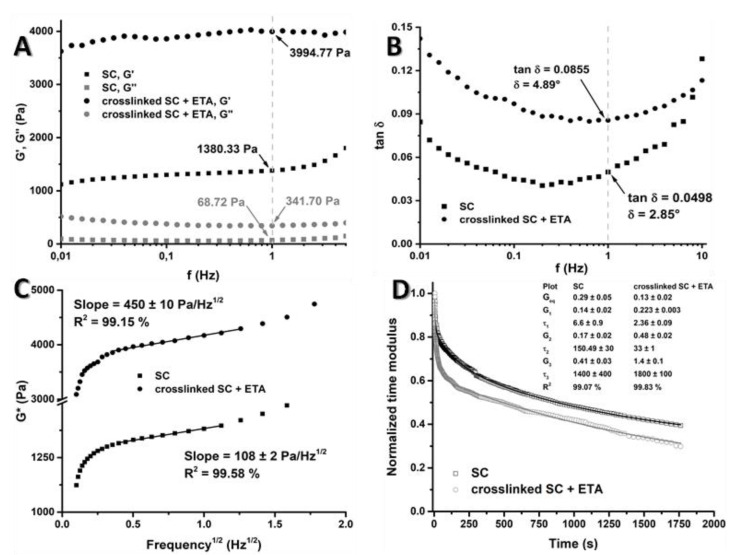
Rheological properties of ETA scaffolds with and without crosslinking. (**A**) Storage modulus (G′) and loss modulus (G″) at 1 Hz. (**B**) Loss factor (tan δ). (**C**) Complex modulus G*. (**D**) Normalized time modulus G(t). *N* = 5 for both samples. Error bars were omitted for clarity. There were statically significant differences among two samples in all the comparisons, *p* < 0.05.

The storage modulus (G′) of a material is defined as the ratio of the elastic (in-phase) stress to strain. It is related to the material’s ability to store energy elastically. Similarly, the loss modulus (G″) is the ratio of the viscous (out-of-phase) component to the stress, and is related to the material’s ability to dissipate stress through heat [30]. Figure 4A shows the results of both parameters obtained at 1 Hz: G′ = 1380.33 Pa and G″ = 68.72 Pa for the scaffolds without treatment, and G′ = 3994.97 Pa and G″ = 341.70 Pa for the crosslinked scaffolds. This provided information about the prominent elastic properties of the scaffolds, because G’ was higher than G″ in both scaffolds. This characteristic is very useful for biomaterial that is being applied in the articular cartilage tissue of the knee due to the cyclic stresses during walking that affect that specific area. Both parameters were higher in the treated scaffold than in the non-treated scaffold. This is because the crosslinking process makes the scaffold more resistant to stress and gives it a greater ability to store energy elastically and dissipate stress through heat.

Comparing these biomaterials with those of previous research [18], the storage moduli of the non-treated scaffold (1.4 kPa) and the scaffold treated with EDC/NHS (3.4 kPa) at 1 Hz were lower than in the scaffold crosslinked with glutaraldehyde (15 kPa). The loss moduli exhibited the same behaviour: the loss moduli of the non-treated material (0.07 kPa) and the material crosslinked with EDC/NHS (0.3 kPa) were lower than that of the material crosslinked with glutaraldehyde (1.4 kPa). Nevertheless, there must be a compromise between the mechanical properties of biomaterials and their biocompatibility. Thus, crosslinking agents, such as EDC/NHS, are more commonly used due to their higher solubility and lower cytotoxicity. It is also valid to highlight that the parameters measured and evaluated are adequate for the use of these scaffolds in implant sites with high cyclic load.

The loss factor (tan δ) is defined as the ratio of G″ to G′. For a viscoelastic material, the phase angle for the strain is between 0° and 90°. For a more elastic material, δ ≈ 0°, and for more viscous materials, δ = 90°. Figure 4B visualises the pronounced elastic character of both materials. The non-treated specimen (δ = 2.85°) had higher elasticity, as evidenced by a lower δ value; however, even when the material was crosslinked, its elastic character was not significantly decreased (δ = 4.89°). Comparing this parameter with that of a similar material crosslinked with glutaraldehyde (δ = 5°) [18], it can be seen that despite the increase in this value, there were no significant differences and the scaffold maintained its elastic character.

The dynamic or complex shear modulus G* (Figure 4C) depends on two parameters: G′ and G″. For both biomaterials, there is an early nonlinear region of the curve at low frequencies (<0.25 Hz), which depends on the intrinsic (fluid-independent) viscoelasticity mechanisms, and a later linear area at higher frequencies (0.25 Hz < f < 2 Hz), which depends on poroelasticity (fluid-dependent) mechanisms [31,32]. The slope of the curves is related to the permeability of the biomaterials [21]. Thus, the permeability decreases as the matrix becomes more compact. For this reason, a lower slope value will correspond to a higher pore size; in this case, the SC without crosslinking had a lower slope value. This result agrees with the SEM images and the conclusion that the crosslinked specimens are more compact and have less porosity than the non-treated specimens.

A very important rheological parameter is the normalized time or relaxation modulus G(t); this assay is performed by applying a stress until the specimen fails. This type of experiment provides data of all the small transitions in the sample structure and the final break time of the material. The relaxation times τ_1_, τ_2_ and τ_3_ (Figure 4D) give the exact moment of a change in the structure: τ_1_ was the failure in the border between the first and the second layer next to the plate, where the stress was applied; τ_2_ was the failure in the border between the second and the third layer, next to the fixed plate; and τ_3_ was the complete failure of the scaffold.

There was a difference between all relaxation times: In the first stages, the τ_1_ and τ_2_ values for the untreated scaffold were higher than those of the crosslinked scaffold because the elastic character of the untreated scaffold allowed for the rearrangement of the structure between layers. In the last stage, τ_3_ was higher in the crosslinked scaffold, because its more rigid network offered resistance for a longer period before failure occurred. Thus, this scaffold is suitable for supporting the cyclic stresses that take place in the knee.

### 3.4. Study of the ETA Binding to the Polymeric Chain in the Scaffold Structure

The chemical bonds between ETA and the polymers in the scaffold could not be estimated by spectroscopic techniques because this protein contains 934 amino acids and the change in functional groups cannot be detected.

In this direction, an immunostaining assay was performed. The assay was completed by staining the ETA using a primary and a secondary antibody with attached fluorophore groups, which are detectable by fluorescence microscopy.

**Figure 5:**
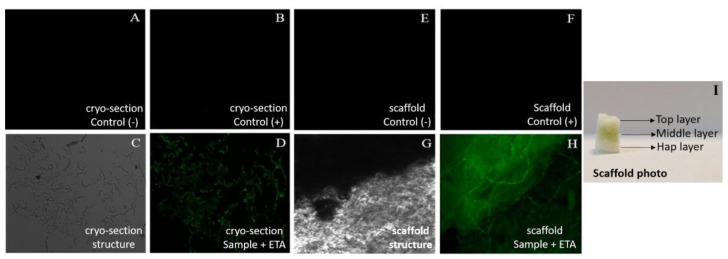
Fluorescent micrographs. (**A**–**D**) Cryosections of 10 µm and (**E**–**H**) scaffold of (3mm × 3 mm × 1 mm) at 5× magnification. (**A**) Negative control, cryo-section of the scaffold without ETA and with primary and secondary antibodies. (**B**) Positive control, cryo-section + ETA without the primary antibody. (**C**) Cryo-section structure. (**D**) Sample, cryo-section + ETA with antibodies. (**E**) Negative control, 3D scaffolds without ETA with antibodies. (**F**) Positive control, 3D scaffold + ETA without antibodies. (**G**) 3D scaffold structure. (**H**) Sample, 3D scaffold + ETA with antibodies. (**I**) Scaffold photo with every layer specified.

The scaffold was first cut into cryosections, stained with both antibodies and later checked using a fluorescence microscope, as shown in Figure 5A–D. The fluorescence was clearly visible as green in the sample (5D). This shows the binding of ETA to the polymeric structure, because it was still in the scaffold after several washes and treatments. To corroborate the results obtained in the slices, the same procedure was performed for the 3D biomaterial. As shown in Figure 5E–H, the sample (5H) had the same outcomes but with a higher signal of fluorescence. This confirms the bond between the ETA and the polymeric chain of the scaffold.

### 3.5. Cell Viability and Proliferation

**Figure 6:**
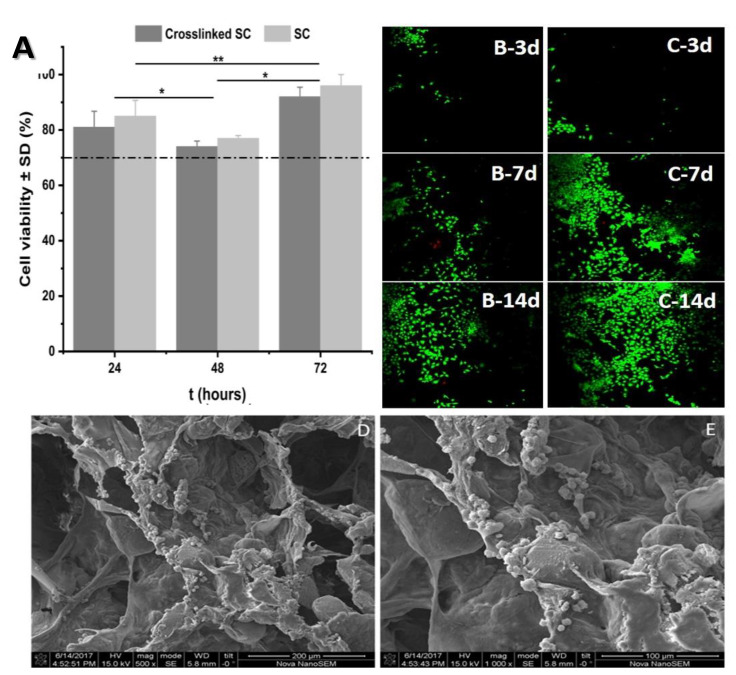
Cell viability assay results. (**A**) Graphic of MTS assay of scaffold with and without crosslinking at 24, 48 and 72 h. Confocal micrographs of the LIVE/DEAD^®^ assay for (**B**) crosslinked and (**C**) non-treated scaffold at 3, 7 and 14 days of culture. Green—live cells; red—dead cells. (**D**) SEM of a crosslinked specimen section at 7 days of culture and (**E**) the specimen at higher magnification. There were non-significant differences at 24 and 48 h. Asterisks indicate significant differences in Student’s t-test, * *p* < 0.05 and ** *p* < 0.01.

The cell viability of the chondrocytes was analysed using an MTS assay, as described in the Materials and Methods section. The percentage of live chondrocytes in the presence of crosslinked and non-crosslinked scaffolds at 24, 48 and 72 h is shown in Figure 6A.

At all-time points, the cell viability was higher than 70%. This result indicates that both materials are non-cytotoxic, according to the ISO 10933-5-2009 [33]. As shown in the bar graph, there was no significance difference in the cytocompatibility of both materials, although the crosslinked scaffold had a slightly lower percentage of live cells at all timepoints; this may have been because small amounts of the crosslinking agents (EDS and NHS) may have remained despite several washes. There was a statistically significant difference between 48 h, 24 and 72 h; however, there was no significant difference between 24 and 72 h.

The proliferation of chondrocytes was studied using a live/dead assay. The treated and non-treated specimens in contact with the cells (Figure 6B,C, respectively) were observed by fluorescence microscopy at 3, 7 and 14 days (from top to bottom). The live cells are shown in green and dead cells are shown in red.

It can be noted by simple inspection that there is a smaller number of cells in Figure 6B, indicating that the proliferation of the chondrocytes was lower in crosslinked specimens, compared to the non-treated specimens (Figure 6C). This is understandable, as there may have been a small amount of EDC and NHS remaining in the polymeric network despite several washes before the study. Nevertheless, there was an increase in the live cells population and there were very few dead cells, indicating that both biomaterials allowed for the proliferation of the cells in vitro.

Figure 6D (SEM of a scaffold section at 7 days) and Figure 6E (higher magnification) show the distribution of the cells not only on the borders of the pores but also inside them. The results showed that the scaffold allowed and promoted the proliferation and homogenous distribution of the chondrocytes through the whole material. The homogeneity of the seeding method has been studied in previous research [16].

This porous scaffold will be grafted next to subchondral bone. In this direction, previous studies have tested the osteogenic character of its calcified layer and the cytotoxicity of the biomaterial in osteoblastic cells [16]. This research demonstrated the osteogenic potential of the calcified layer and the non-cytotoxicity of the porous bioscaffold in osteoblasts.

### 3.6. In Vitro ETA Controlled Release Study

The incorporation of a system (scaffold and anti-inflammatory drug) in the damaged cartilage area has many advantages, such as a longer duration of action by sustained release of the drug and lower plasma levels with less toxic and less systemic exposure, leading to a reduction of side effects and injection frequency. In this direction, an in vitro release study of ETA was developed for the crosslinked and non-crosslinked scaffolds to evaluate the influence of the crosslinking process on the release end time and kinetic of the process.

**Figure 7:**
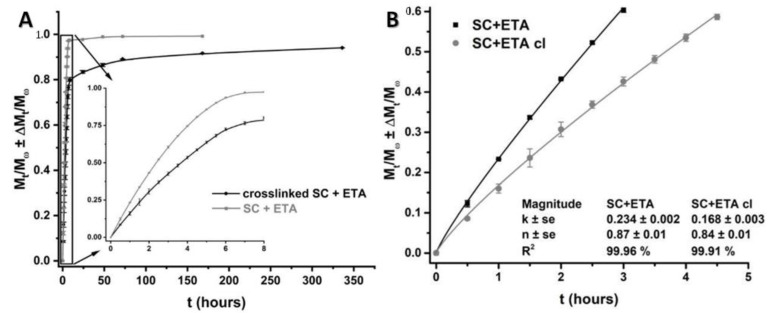
In vitro ETA controlled release study on scaffolds with and without crosslinking. (**A**) Drug release profiles. (**B**) Fitting the Peppas power law (*N* = 5).

Figure 7A shows the profiles in grey (scaffold and ETA) and black (crosslinked scaffold and ETA). The non-treated material had the maximum percent of release at 7 h (93%), while the crosslinked material released ETA for 7 days (95%). These results are very promising for damaged cartilage tissue because this system can reduce the frequency of drug administration in the body.

It can be seen that the values of n (0.87 and 0.84 for the non-crosslinked and crosslinked scaffolds, respectively, Figure 7B) support the theory of the simultaneous control of drug delivery by diffusion and polymer chain relaxation, although control is more displaced toward the latter. In fact, the solubility coefficient of the drug (part of the k coefficient) decreased by about 30% due to the crosslinking process in the scaffold.

### 3.7. In Vitro NO, IL-6 and TNF-α Production

Nitric oxide (NO) produced by the cartilage and synovial membrane is implicated in the pathogenesis of osteoarthritis (OA) and rheumatoid arthritis (RA). In inflamed joints, NO is synthesized in response to proinflammatory cytokines and it is involved in joint destruction [34]. In murine macrophage RAW 267.4 cells, lipopolysaccharide stimulation induces the production of neurotransmitters, such as nitric oxide, inflammatory mediators and pro-inflammatory cytokines.

ETA is an inhibitor of TNF-α [35] and other cytokines. It also reduces NO production, which plays an important role in the inflammatory process of articular cartilage. For this reason, the production of nitric oxide, interleukin 6 and tumour necrosis factor alpha in vitro provides the possibility for the evaluation of the anti-inflammatory effect of ETA occluded in the 3D scaffold grafted in an osteoarthritic knee.

**Figure 8:**
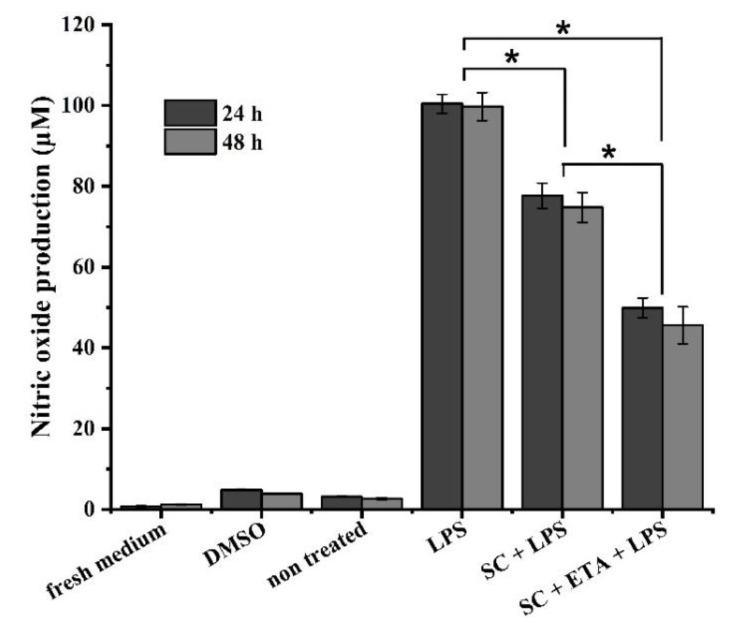
In vitro nitric oxide production with LPS-treated RAW 267.4 cells. There were non-significant differences (24 and 48 h). Asterisks indicate significant differences in Student’s *t*-test, * *p* < 0.05.

Figure 8 shows the production of nitric oxide at 24 and 48 h after the activation of the RAW cells. There was no significant difference between the time points; however, there were differences between the samples. Compared to the negative (non-treated) and positive control (LPS), the results were very encouraging: the scaffold with LPS diminished NO production. This is favourable because it indicates that the inflammation was reduced due to the presence of hyaluronic acid in the 3D biomaterial. However, the scaffold with ETA and LPS decreased the production of NO more than the sample without ETA. This shows that this drug has a high anti-inflammatory effect on the damaged cartilage tissue.

Cytokines are regulators of host responses to infection, immune responses, inflammation and trauma. Some cytokines act to make disease worse (proinflammatory), whereas others serve to reduce inflammation and promote healing (anti-inflammatory) [36]. The production of proinflammatory cytokines, such as IL-6 and TNF-α, is initiated by organisms themselves (phagocytosis) or by soluble products: for example, the lipopolysaccharide (LPS) endotoxins of Gram-negative bacteria; the protein exotoxins of Gram-positive bacteria; and cell-wall glycopeptides, such as teichoic acids and muramyl peptides [37].

**Figure 9:**
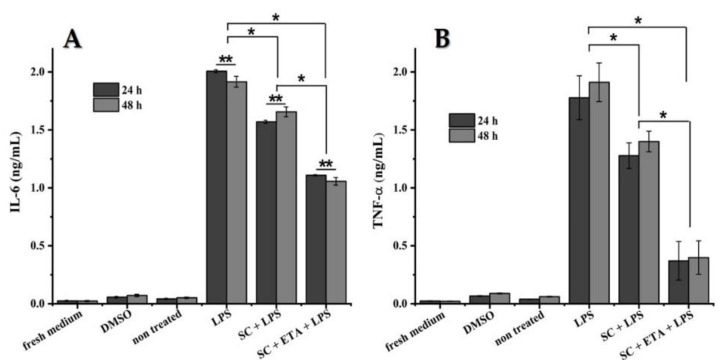
In vitro assay with LPS-treated RAW 267.4 cells. (**A**) Interleukin 6 production and (**B**) tumour necrosis factor alpha production. For (**B**), there were non-significant differences (24 and 48 h). Asterisks indicate significant differences in Student’s t-test, * *p* < 0.05 and ** *p* < 0.01.

In this assay, IL-6 and TNF-α were produced by activating macrophages with LPS in scaffolds with and without ETA to evaluate the efficacy of this drug. The bar graphs of IL-6 and TNF-α production (Figure 9A,B, respectively) clearly demonstrate that ETA considerably diminished the production of both proinflammatory cytokines, with significant differences between the values of the positive control (LPS), the scaffold without anti-inflammatory effect and the one with the drug occluded. TNF-α production was diminished by almost five times by the ETA anti-inflammatory effect, while IL-6 was decreased by just two times. There was no significant difference between the values of each sample at 24 and 48 h.

### 3.8. In Vivo Study of ETA Controlled Release

**Figure 10:**
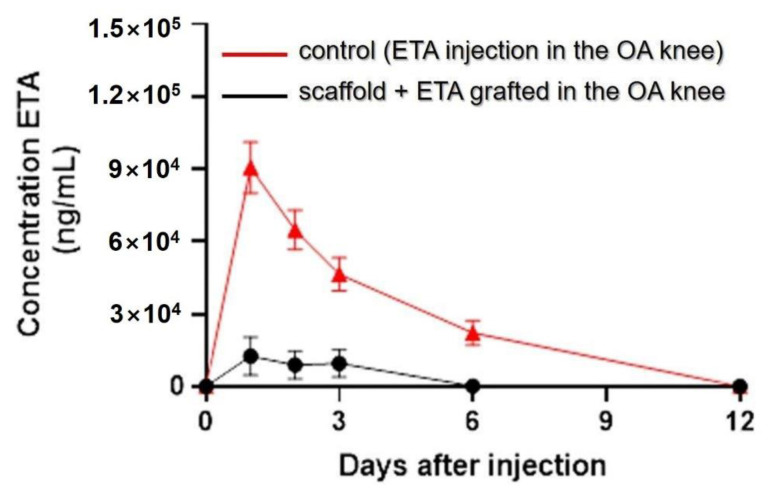
In vivo ETA controlled release in 10 µL of blood collected from mice at 0, 1, 2, 3, 6, 9 and 12 days after operation. The ETA concentrations were determined by ELISA. The red curve represents the control (ETA injection in the OA knee) and the black curve represents the scaffold + ETA grafted in the OA knee.

The controlled release of ETA into the blood from the grafted crosslinked scaffold was evaluated in mice. Serum was extracted from blood collected at certain time points, and an ELISA assay was performed to determine the ETA concentration. Figure 10 shows the comparison between these concentrations and the control (ETA injection concentrations).

The ETA in vitro controlled release profile was not as long as the in vitro test for the crosslinked material, at 6 and 14 days, respectively. This is because the dimensions of the biomaterial and the drug percent for the in vitro assay were higher in comparison to those of the in vivo assay. This aids in the understanding of the release mechanisms that occur during this process. Another factor that may have led to the difference in time release is the environment: biological osmosis in vivo could have accelerated the release process, while in vitro, the samples were kept in an oven during the study without any movement.

### 3.9. Imaging, Micro-CT and Histology

**Figure 11:**
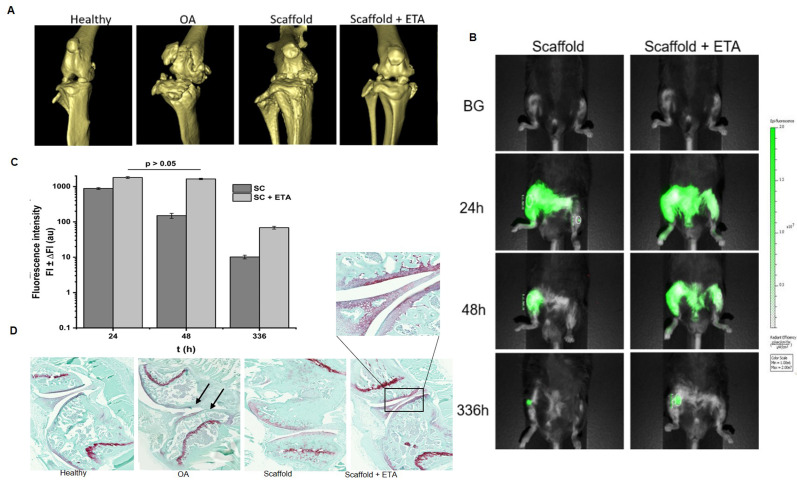
(**A**) Representative Micro-CT of healthy (negative control group), osteoarthritis (OA) knee (positive control group), empty scaffold and scaffold loaded with ETA grafted in the damaged area. (**B**) Near-infrared (NIR) imaging of a scaffold with and without ETA grafted in the right knee joint of mice with chemically-induced osteoarthritis before grafting (BG), and at 24 h, 48 h and 336 h post-grafting. (**C**) Bar graph of NIR fluorescence intensity over time. (**D**) Representative images of empty scaffold and scaffold with ETA ex vivo, with histological staining using safranin-O and fast green. From left to right: healthy knee (negative control), OA knee (positive control), representative picture of OA knee grafted with empty scaffold and scaffold + ETA.

It is well known that osteoarthritis causes bone structure to change significantly [38]. To address this, micro-CT scans were used to visualize the bone of 3D, healthy and osteoarthritic mice. The stages of damage in the articular cartilage were compared after the scaffold (with and without ETA) was grafted into the knees of the mice (Figure 11A). The results after four weeks of study were encouraging as there was a notable difference between the OA knee and the knees with both grafted biomaterials (with and without drug). There was a slightly increased reduction in joint degeneration in the scaffold with integrated ETA.

The micro-CT imaging technique (Figure 11B) was used to follow the grafted scaffolds inside the OA knees of the mice before grafting, and 24, 48 and 336 h post-grafting. The fluorescence intensity of NIR inside both materials diminished by 100 times in the scaffold alone, and by 10 times the scaffold with ETA at 336 h. At the end of the study (35 days), there was a green colour in the joint, indicating that there was still some non-degraded material in the damaged area. This result was corroborated by the bar graph of intensity (Figure 11C).

The benefits of incorporating a scaffold system with ETA into an osteoarthritic knee were investigated with an in vivo OA mice model, in which the mice were subjected to osteoarthritis simulation induction with collagenase, then treated with the three-layer porous scaffolds; finally, the histological analysis of the knees using safranin-O and fast green staining was performed. The safranin-O and fast green staining assay of the knees was performed to visualize the characteristics of the articular cartilage (Figure 11D). The healthy knee had a continuous tidemark (in white), and its cartilage surface was regular, the cartilage of the OA knee without biomaterial was degenerated and its tidemark (black arrows) was disrupted, while the OA knee with grafted scaffolds had less damage in the articular cartilage compared to the OA knee without the scaffold; the tidemark in both cases was continuous. These results reveal that the presence of this material improved the health of the mouse knee with OA cartilage, indicating the SC and ETA system had better outcomes than the empty SC.

## 4. Conclusions

We incorporated an anti-inflammatory drug into an implantable porous tri-layer scaffold for cartilage repair. This biomaterial was based on collagen and chitosan. In one of the three layers, it contained hydroxyapatite as a bioactive component. The in vitro, in vivo and physicochemical results suggest that the combination of this biomaterial with ETA is appropriate for chondrocyte growth and proliferation, non-cytotoxic and suitable for in vivo use for at least 5 weeks. However, the search for the ideal scaffold continues. In this direction, the system could be improved by incorporating growth factors before pursuing late preclinical development.

## Data Availability

The data presented in this study are available on request from the corresponding authors.
